# A Cyst of the Cerebellum1Read before the Buffalo Academy of Medicine, March 21, 1892.

**Published:** 1893-07

**Authors:** Linus J. McAdam

**Affiliations:** 88 Austin Street; Buffalo, N. Y.


### A CYST OF THE CEREBELLUM.[1]

By LINUS J. McADAM, M. D., Buffalo, N. Y.

In reporting this case I do not wish to convey the idea that it is so very different from other cases of the same nature, but the definiteness of its symptoms, the rapidity of its course, its fatal termination, and the fact that we were able to get an autopsy and secure the specimen, warrant its publication.

Jacob Mc., aged 36; millwright by trade; of good family history. On October 23, 1892, he called at my office, complaining of dizziness, nausea, and a pain in the back of his head, and that morning while yet in bed he had a strange feeling come over him, as though everything was going round and seemed to turn him partly round in bed. For a number of days he had noticed, while working in a stooping position, that he would suddenly lose his balance and butt his head against whatever might be in the way. Upon examination I found him with a furred tongue, sallow complexion, yellow conjunctivæ, loss of appetite, temperature sub-normal, ninety-eight degrees; high-colored urine, and constipation, together with the other symptoms which I have just mentioned.

From this I drew the conclusion that there was a torpidity of the secretions, and prescribed accordingly, blue mass, followed in the morning with Rochelle salts and a bitter tonic, asking him to report the results, which he did in two days, with very little change in his condition. Without changing my diagnosis I gave him more hepatic stimulants and waited developments. After two days he returned complaining of a good deal of thirst, and upon inquiry he was passing more than the normal amount of urine; requesting him to save the urine passed in twenty-four hours, I found he was passing from sixty to seventy ounces daily, which upon chemical analysis contained quite a quantity of sugar. Thinking, perhaps, this was the cause of the symptoms, I put him on mineral waters with Rhus aromatica, and in a few days the sugar disappeared. Inquiring more closely into the history, I

1. Read before the Buffalo Academy of Medicine, March 21, 1892.

found that on one occasion while down the river fishing, he had experienced a sort of sunstroke, and upon inquiry I found that he had ringing in the ears and tingling in his fingers, and when his eyes were closed there would be sparks of light passing before them. The former symptoms still persisting, with these new facts in my possession I diagnosticated a sub-acute basilar meningitis. My reasons for calling it basilar were : (1st) That the symptoms pointed to the base ; (2d) that there were no mental symptoms, the speech, hearing, and sight being unaffected. Changing my tactics I kept up the cathartics and put him upon a mixture of bromide and ergot, which seemed to relieve him, until October 31st, when I noticed that if his attention was attracted while he was walking he would swagger to the right. Having inquired as to any specific history I thought that perhaps he did not wish to admit. I began to think that there might be a gumma and placed him upon the iodides, which after a persistent use for two or three weeks failed to give any results. The symptoms becoming more grave, I consulted Dr. Crego on December 5, 1892, who, after hearing the history, and upon examination, pronounced it a lesion of the base, and also stated that it might be due to one of several conditions of which he mentioned, an occlusion of the post-cerebral artery, gumma, tumor or softening, and recommended larger doses of the iodide with galvanism. On December 22, 1892, I again consulted him. The tendency of the patient to go to the right and the marked atoxic gait were unmistakable, and the lesion was located in the cerebellum ; still continuing the iodide, we gave mercurial inunctions from time to time without any results. On January 17th, Dr. Wm. C. Krauss was called in consultation, and made a more positive diagnosis of a neoplasm of the cerebellum. Anxious to follow the case through, although it drifted into other hands who gave them more hope than I could, I made occasional friendly calls, until the night of March 15th, when I was called only to find what we had anticipated. To my surprise he died without a struggle. With some difficulty I obtained the autopsy, and the specimen which demonstrated that the diagnosis was correct, namely : A cyst of the right lobe of the cerebellum.

88 AUSTIN STREET.

